# Murine leukaemia virus expression in the AKR following thymectomy.

**DOI:** 10.1038/bjc.1975.248

**Published:** 1975-10

**Authors:** M. Tuffrey, P. Crewe, L. Dawson, J. Holliday, R. D. Barnes

## Abstract

Thymectomy effectively prevents the development of spontaneous lymphoma in the AKR but how this effect is achieved remains to be determined. One possible mechanism, namely suppression of genomic expression of the oncogenic murine leukaemia virus now seems unlikely since levels of the group specific MuLV antigen were in comparision with their sham operated controls unaltered in both neonatally and adult thymectomized AKR.


					
Br. J. Cancer (1975) 32, 465

MURINE LEUKAEMIA VIRUS EXPRESSION IN THE AKR FOLLOWING

THYMECTOMY

M. TUFFREY, P. CREWE, L. DAWSON, J. HOLLIDAY AND R. D. BARNES

From the Clinical Re8earch Centre, Harrow, Middle8ex

Received 2 May 1975. Accepted 20 June 1975

Summary.-Thymectomy effectively prevents the development of spontaneous
lymphoma in the AKR but how this effect is achieved remains to be determined.
One possible mechanism, namely suppression of genomic expression of the onco-
genic murine leukaemia virus now seems unlikely since levels of the group specific
MuLV antigen were in comparison with their sham operated controls unaltered in
both neonatally and adult thymectomized AKR.

ALTHOUGH quite clearly involved, the
exact role of the thymus in the develop-
ment of the lymphoma which charac-
terizes the AKR (Furth, Seibold and Rath-
bone, 1933) still remains to be deter-
mined (Miller, 1961). One possible effect of
thymectomy may be to suppress the
titres of murine leukaemia virus group
specific antigens and this has been exam-
ined here in both neonatally and adult
thymectomized AKR.

MATERIALS AND METHODS

Mice.-The derivation of the AKR/CRC
used here has been described previously
(Barnes and Tuffrey, 1974). Initially AKR/J
they were then maintained at the Laboratory
Animal Centre, Carshalton before being
transferred first to the National Institute of
Medical Research at Mill Hill and then to the
Clinical Research Centre. At all times the
colony was maintained by brother-sister
mating. As noted earlier, the AKR/CRC
subline have a high incidence of lymphomata
reaching 100% by 56 weeks of age (Barnes,
Tuffrey and Ford, 1973).

Surgery.-The mice were either thymecto-
mized or sham operated. In both cases the
procedure either took place during the first
24 h of life or at varying intervals from the
age of 4 weeks. Details of the technique of

thymectomy have been described previously
(Tuffrey, Kingman and Barnes, 1973). In
the case of the mice operated on during the
first 24 h of life, hypothermia was employed.
Anaesthesia in adult groups aged between
6 and 9 weeks was achieved using bromethol
(Avertin), 0 01 ml/g body weight i.p. of 2.5%
solution).

Investigation.-Animalswerekilled at vari-
ousintervalsfollowingthymectomy. Success of
thymectomy was confirmed by histological
examination of serial sections prepared from
the mediastinum.

Tissues and serum were obtained at
sacrifice and stored at - 20?C until investi-
gated. The indirect immunofluorescent
absorption technique of Hilgers (Hilgers
et al., 1974) was performed to determine
the gs-antigen titres, but here against fixed
AKR-A lymphoma cells. The AKR-A
lymphoma cell line was originally derived by
Woods et al. (1970) and maintained here at
the Clinical Research Centre by Dr Jennifer
Harvey. In essence, the gs-antigen test
is in 2 stages: first the titration of the
specific anti-MuLV-gs sera against the target
AKR-A lymphoma cells, followed by a second
titration after absorption with soluble anti-
gens obtained in the case of solid tissues
after ultrasonic disintegration. The gs-
antigen titre is expressed as the reciprocal
of the reduction in antibody titre following
absorption (I.F.A.). In all cases coded

Correspondence to: Dr R. D. Barnes, Department of Infant Development, Clinical Research Centre,
Watford Road, Harrow, Middlesex, HAL 3UJ.

466   M. TUFFREY, P. CREWE, L. DAWSON, J. HOLLIDAY AND R. D. BARNES

samples were examined and the results were
then subsequently related to each animal and
to the success of surgery. Some untreated
controls were also included. These were 3
old AKR with lymphomata and some young
CBA/H-T6.

RESULTS

The results of the gs-antigen titres in
the adult thymectomized and sham
operated AKR are shown in Table I.
Thymectomized mice in which a micro-
scopic remnant of lymphoid tissue (prob-
ably lymph node) was subsequently found
on histological examination were main-
tained as a separate subgroup.

The results of the gs-antigen titres
from the neonatally thymectomized and
sham thymectomized AKR are shown in
Table II. The distribution of MuLV-gs
antigen appears in good agreement with
the titres described by Hilgers et al. (1974).
Although we were unable to detect any
MuLV-gs antigen in any of our serum
samples, we were able to detect MuLV-gs
antigen in the spleen, liver and kidney
from one week of age. Highest titres
were usually detected in the spleen samples,
apart from those recorded from control
AKR mice with thymoma (Table III).
This is also in accord with the infectivity
titres recorded by Rowe and Piiicus (1972).
It should be noted that the titres in the
AKR are somewhat lower than those
described by previous workers (Hilgers
et al., 1974). This difference is due solely
to technical variation in determination of
the " end point ".

It is quite obvious from these results
that thymrectomy, whether neonatal or
between 4 and 9 weeks of age, has no
effect upon MuLV-gs titres.

DISCUSSION

The involvement of the oncogenic
virus in the disease of the AKR strain
was clearly established by Gross (1951,
1957). Although the role of the thymus
in the disease process of the AKR remains
speculative, thymectomy at any stage
before the development of a lymphoma
effectively prevents the disease (McEndy,
Boon and Furth, 1944; Law and Miller,
1950). This advantage might act in one
of several ways, one being that thymectomy
might suppress viral gene expression and
its neoplastic sequelae. From the findings
here, this view seems unlikely since com-
parable amounts of MuLV-gs antigen
were found in both sham operated and
thymectomized AKR. These results can
be considered to support Miller's (1960)
work in which he showed that his " leukae-
mic agent" was able to multiply when
transferred through a series of thymecto-
mized hosts. Nakakuki, Shisa and Nishi-
zuka (1967) suggested that the viral con-
centration needed to reach a critical level
to enable initiation of neoplastic trans-
formation, however it would appear that
the protective effect of thymectomy is
not in reducing the MuLV-gs titre and
therefore possibly the level of infectious
virus.

The most obvious interpretation would

TABLE III.-MIuLV-qs Titres on Tissues and Serum           of Leuk-aemic AKR/CRC        and

normal. CBA/H-T6 Mice

Controls (age days)  No. of animals  Spleen Kidney  Liver Thymus Serum     B/M    L/N
AKR* 1                                  8      2       16      16      4      8       16

2                               4       1       8      16      8       8       4
3                               8       2      16      16      8       4      nt

CBA     1 (6) days

2 (9) days
3 (+ 100)

7
4
3

0
0
0

0
'0
0

0
0
0

0
0
0)

0
0
0

nt
nt
nt

nt
nt
nt

Results expresse(l as reciprocal of I. F.A. titres.
* Leuikaemic.

MURINE LEUKAEMIA VIRUS EXPRESSION IN AKR FOLLOWING THYMECTOMY 467

C)   C

C)

0

0

t o _ o o o    o   'o.oo   o

Co EO  " to t 00>0       00000000000 ?

CO ,u  S

0?=s>>Xt

;a  e  o >fcq    --     ---__-00

I.      oem   oWo

C)

; Cxttct tccuo

EH  a X o Q~~~~~~bo

;4 o ;

468    M. TUFFREY, P. CREWE, L. DAWSON, J. HOLLIDAY AND R. D. BARNES

01I 000000

0010--

_I C4  _ _ 0  o   CO

_   __2

0 0000 0o
o1 oooo10 oo
o~~~ o       C C

oP~~~~~~~~q qId qa

0

01

P-4- 0  --  0)  I

01 -4 I"   aq Co 0

0 - -  0 - -->C)0

O O O O C) 0 O

__

00

_   _ 0

__01

0 o o o
o o o o
00 00 cs

oe- -o o

000

t t e10e

10 0 1 1   1 -Z

0 -- 0 0 0  ___

0o 0    0 o   oo

___

ooooo_oo

0_

0 o

00(C

o o

o o

0 o

?4?4

0

Co
04

00

04
0

to

b~

0
Co
0
X o 6

00S

0

Co
eO0 0

0 ?4?4?4 ? ?

0
Co
C)
0
0
0

04
0

u       I

0H

CS

0_

0

MURINE LEUKAEMIA VIRUS EXPRESSION IN AKR FOLLOWING THYMECTOMY 469

-0 _ o o o C o C

O    O  0 O  0- -  ese

Il  l   I  I   I   I   I  I   I   I   I   I   I
III     III I I I

I e- _          o-_o   aq c q

I 0  1 000

C)
D

k

C3)

> _oom ,*N
C)
IbC$

m I

4

000
IC$

Sb

-00

00

o I

0 O 0
;1

C)

A12 .d4 0>-
CC4

-0 0000      0-- --00 C

*     *

Ci   I I C   I  I  I  I  I I

*       *

r-A

cs cq cs cs eq cq aq cq cs

ooo)   000000000000010)o oo  I  0  oo_o
_Oo ooooooo___o_oolo ooCO

oo  o o o _  o  s _4 C)  o o o  o) o  oO

C)   O
Ro&

* *
- c

`Ca,
m

Sb

0

C)

a,

C)

02
0

m
I"
0

OC 1 0 -
Sb   Sb

10
p d

0 Ca
Sb

0

0 CM

Cb

04
C)
0

CC

. .-

.

0
4:)
0
T$
w
0
C)
0f

.-

C)

Pq

0
C0

C)

C.')

xo

VL

I_ _ I  *  *

II IIIII II 1111   &Kt& J  'II

* *  *-** ** *- * * * * --* * *  * *

oo_  -  -----~0 ______ C C I IC)1C)IcC)

0 -C)  I-   ~-

as     g  -

470   M. TUFFREY, P. CREWE, L. DAWSON, J. HOLLIDAY AND R. D. BARNES

be that the target cells for neoplastic
conversion  are  removed. However,
Miller (1961) clearly demonstrated that
by grafting CBA/H-T6 and AKR thymuses
into thymectomized (AKR x T6) Fl, the
resulting leukaemlic cells were of host
origin which suggested that the "thvmic
influence responsible for leukaeinic change
was a non-cellular indirect one".

It is now established that AKR mice
are not permanently tolerant to MuLV
since anti-MuLV gs antibodies are formed
and can be demonstrated as antibody-
antigen complexes in the kidneys from
about 3 months of age (Oldstone
et al., 1972). Since MuLV-gs antigen
levels are unchanged in non-leukaemic
thymectomized AKR mice, we are at
present looking for antibodies in these
animals to see whether tolerance to the
virus has been maintained and whether
this is in fact related to tumour develop-
ment a view that has been suggested
earlier (Barnes and Tuffrey, 1974).

REFERENCES

BARNES, R. D. & TUFFREY, M. (1974) Lymphoma

Susceptibility of the AKR Mouse Strain Acquired
Before the Stage of Implantation. Br. J. Cancer,
26, 400.

BARNES, R. D., TUFFREY, M. & FORD, C. R. (1973)

Suppression of Lymphoma Development in
Tetraparental AKR Mouse Chimaeras Derived
fiom Ovum Fusion. Nature, New Biol., 244, 282
FTJRTH, J., SEIBOLD, H. R. & RATHBONE, R. R. (1933)

Experimental Studies on Lymphomatosis of Mice.
Am. J. Cancer, 19, 521.

GROSS, L. (1951) Spontaneous" Leukaemia Devel-

oping in C3H Mice following Inoculation in Infancy,
with AK-leukemic Extracts or AK-embryos.
Proc. Soc. exp. Biol., N. Y., 76, 27.

GROSS, L. (1957) Studies on the Nature and Bio-

logical Properties of a Transmissible Agent causing
Leukemia following Inoculation into Newborn
Mice. Ann. N.Y. Acad. Sci., 68, 501.

HILGERS, J., DECLEVE, A., GATESLOOT, J. & K U'LAN

H. S. (1974) Murine Leukaemia Virus Group-spec-
ific Antigen Expression in AKR Mice. Cancer
Res., 34, 2553.

LAW, L. W. & MILLER, J. H. (1950) The Influence of

Thymectomy on the Incidence of Carcinogen-
induced Leukemia in Strain DBA Mice. J. natn.
Cancer Inst., 11, 425.

MCENDY, D. P., BOON, M. C. & FURTH, J. (1944)

On the Role of Thymus, Spleen and Gonads in
the Development of Leukemia in a High-Leukemia
Stock of Mice. Cancer Res., 4, 377.

MILLER, J.F.A.P. (1960) Recovery of Leukaemo-

genic Agent from Non-leukaemic Tissues of
Thymectomised Mice. Nature., Lond., 187, 703.
MILLER, J. F. A. P. (1961) Analysis of the Thymus

Influence in Leukaemogenesis. Nature, Lond.,
191, 248.

NAKAKUKI, K., SHISA, H. & NIsHIzUKA, Y. (1967)

Prevention of AKR Leukemia by Thymectomy
at Varying Ages. Acta Haemat., 38, 317.

OLDSTONE, M., TISHON, A., TONIETTIl, C. & DIXON, F.

(1972) Immune Complex Disease Associated with
Spontaneous Murine Leukaemia Incidence and
Pathogenesis of Glomerulonephritis. Clin. Imrnu-
nol. Immrunopath. 1, 6.

ROWE, W. P. & PiNcus, T. (1972) Quantitative

Studies of Naturally Occurring Murine Leukaemia
Virus Infection of AKR Mice. J. exp. Med., 135,
429.

TUFFREY, M., KINGMAN, J. & BARNES, R. D. (1973)

The Failure to Transfer the NZB Disease follow-
ing Implantation of NZB Thymus containing
Chambers. Eur. J. Immunol., 3, 519.

WOODS, A., WIVEL, N. A., MASSICOT, J. G. &

CHIRIGOS, M. A. (1970) Characterisation of a
Rapidly Growing AKR Lymphoblastic Cell Line
Maintainiing Gross Antigens and Viral Replication.
Cancer Res., 30, 2147.

				


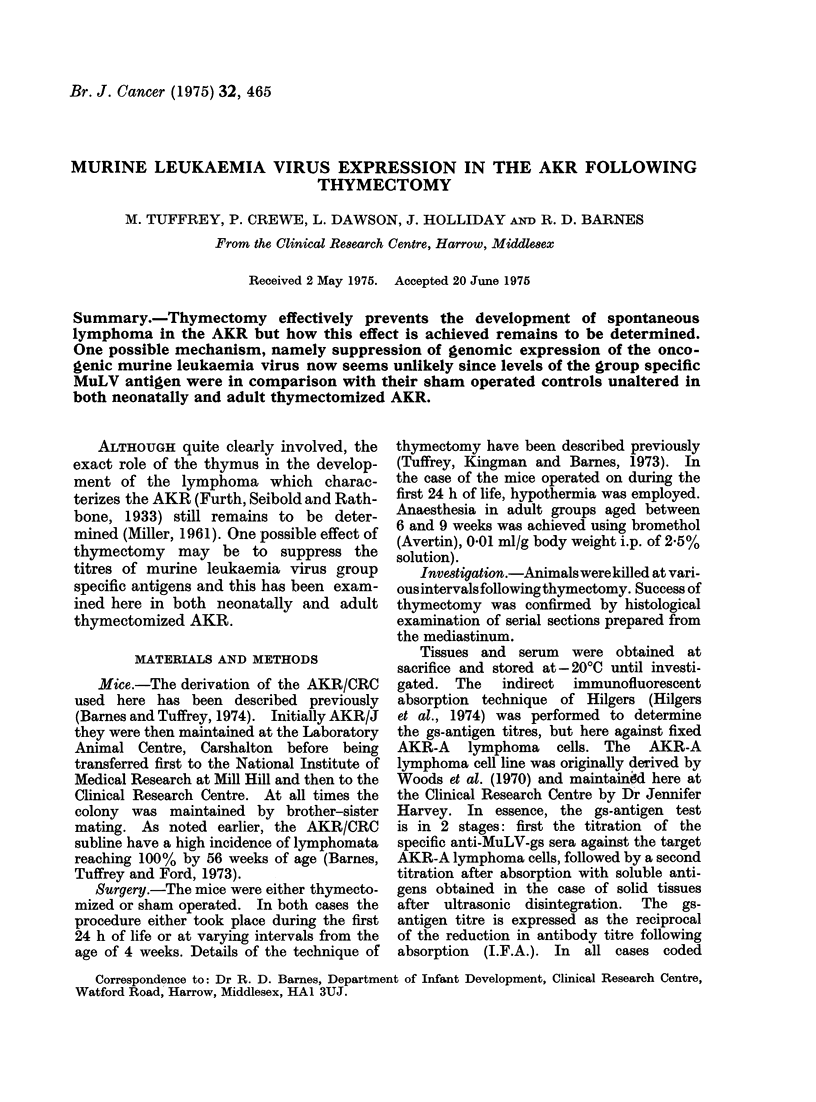

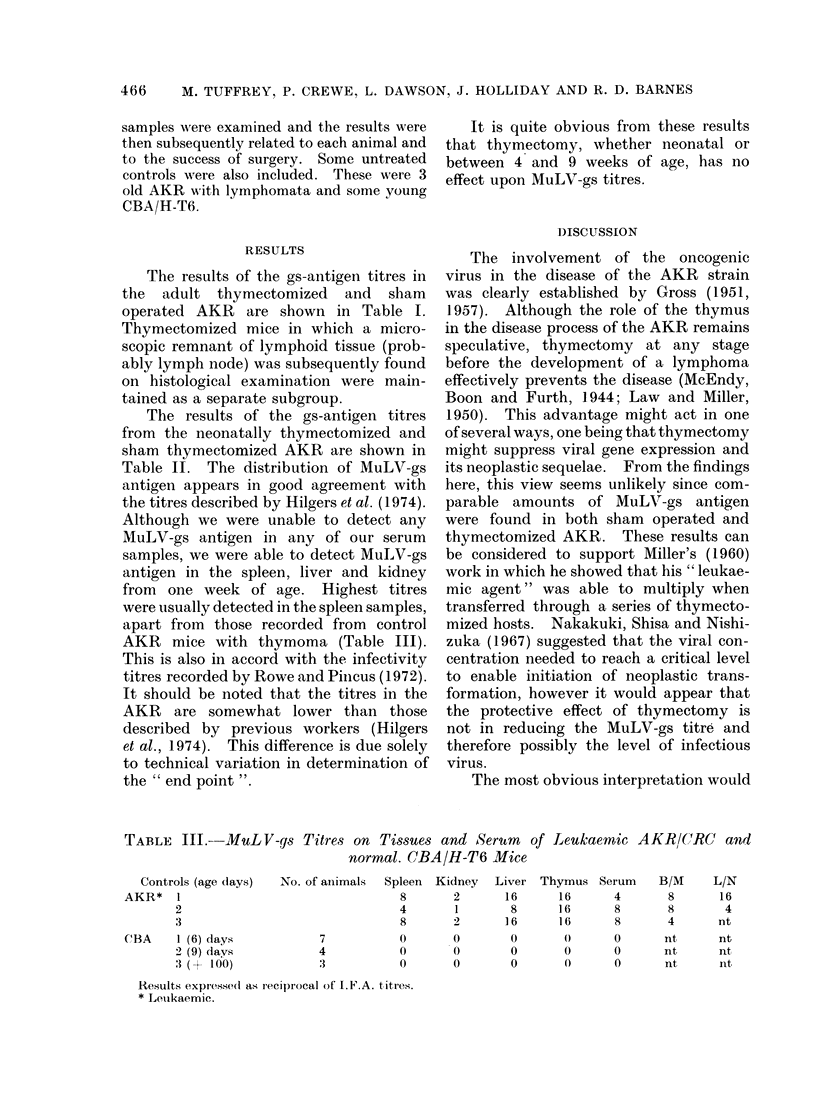

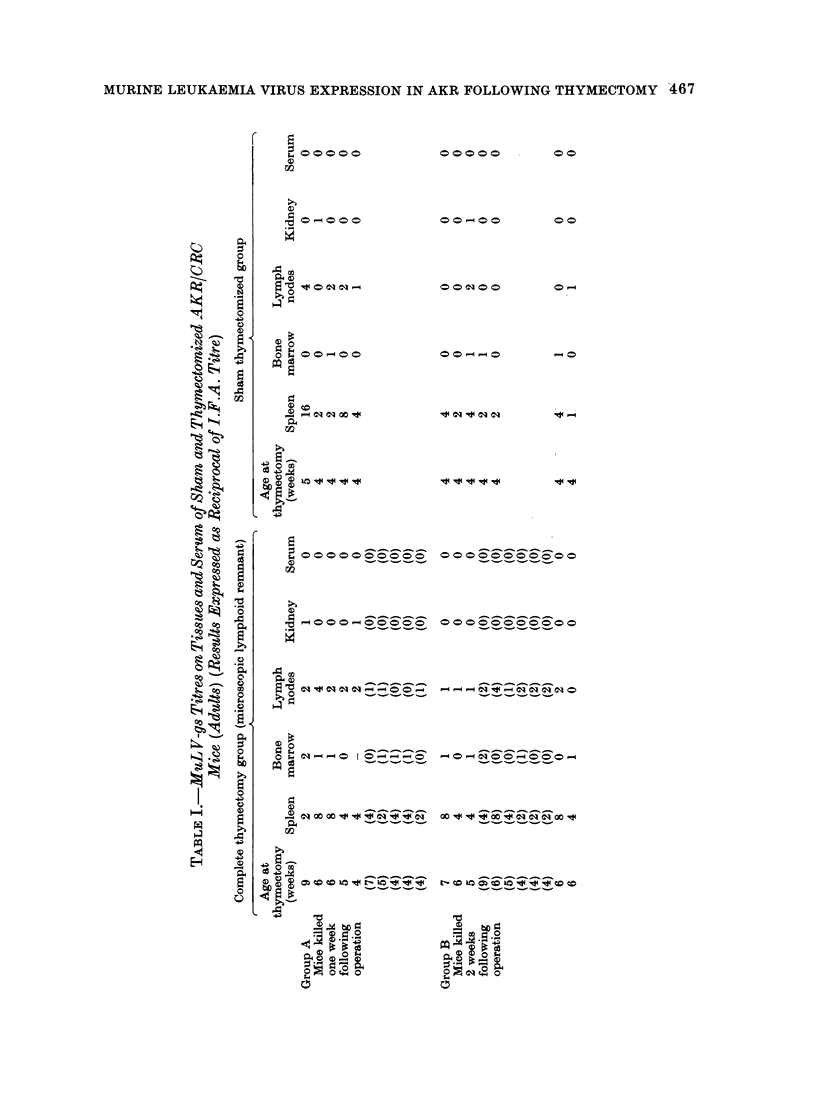

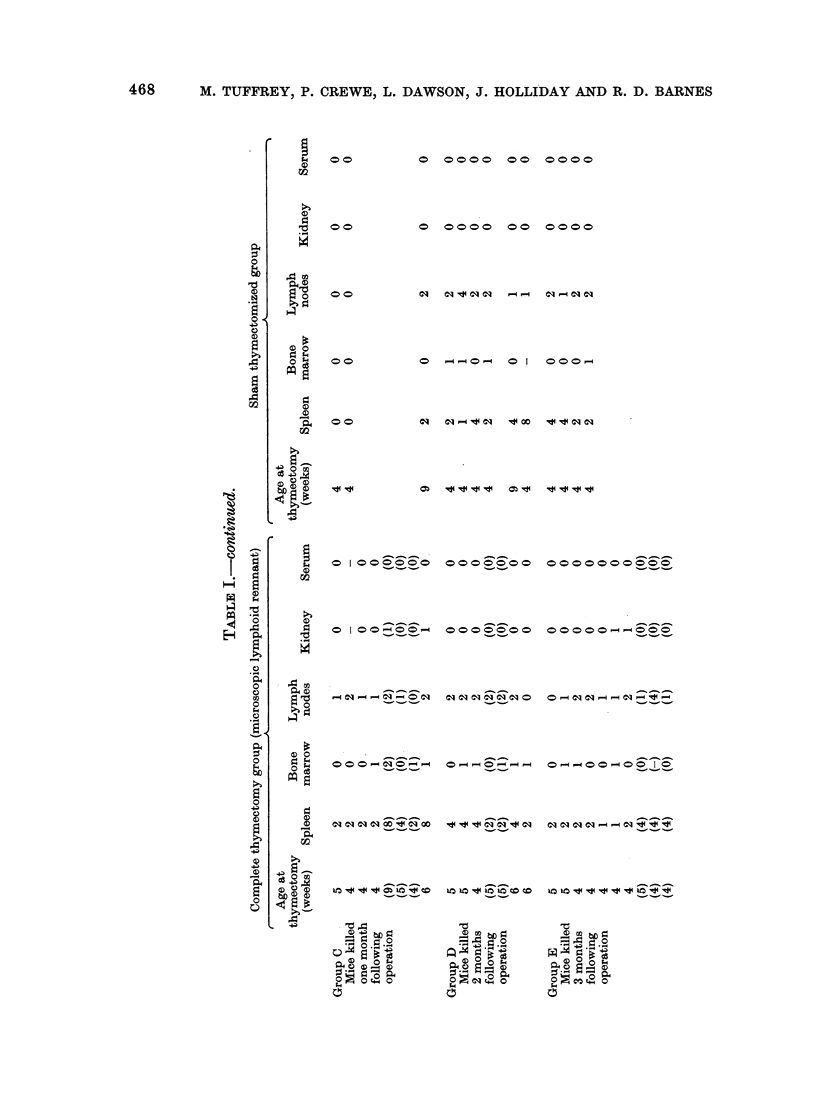

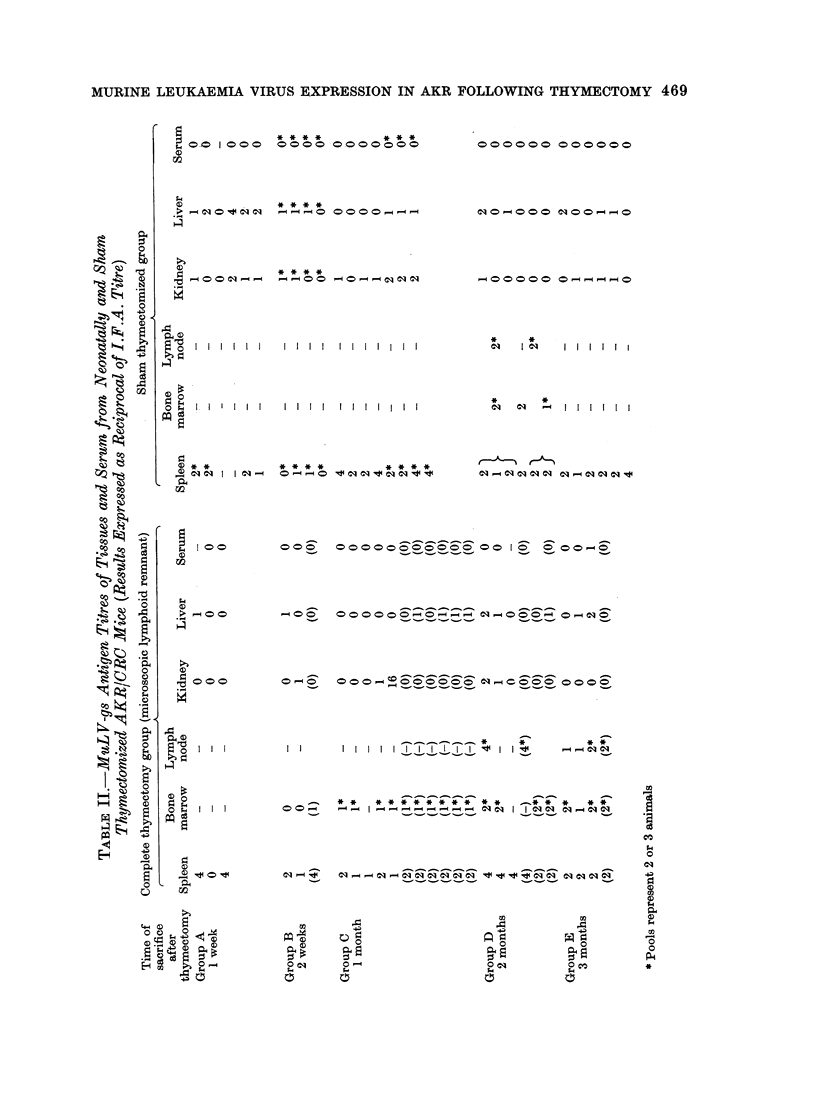

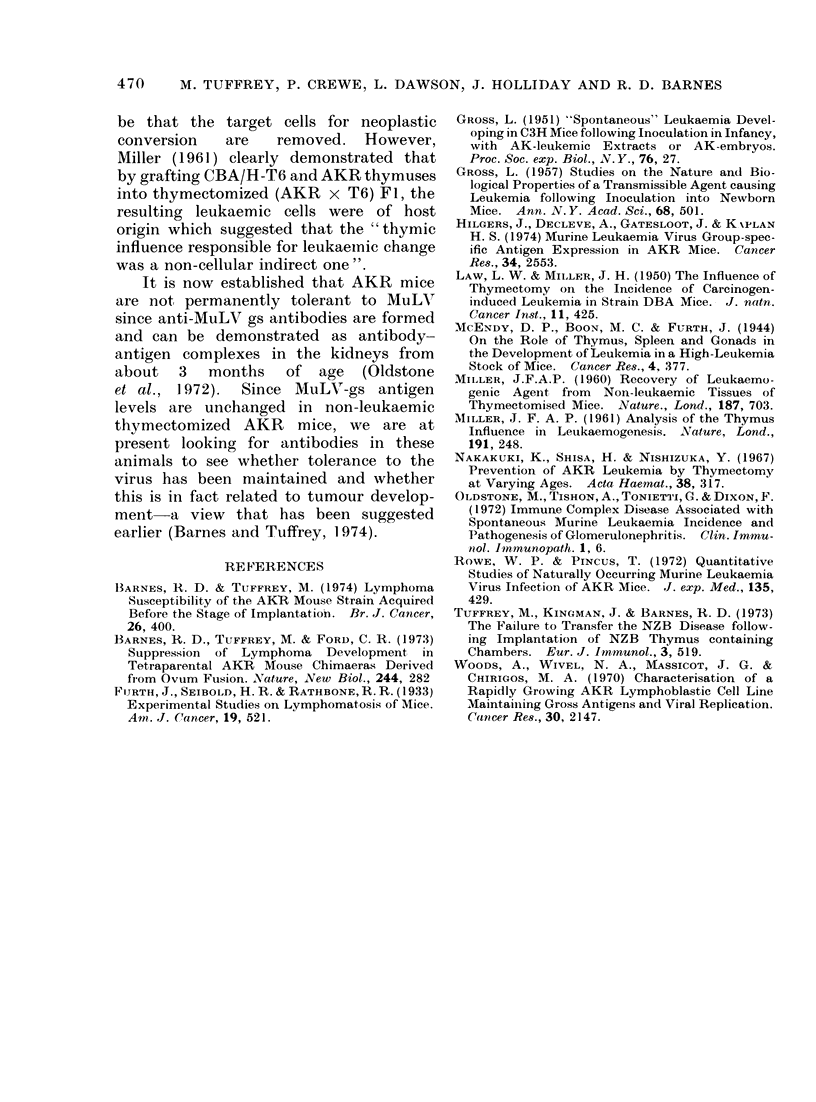

